# Utility of radiographic measurements to predict echocardiographic left heart enlargement in dogs with preclinical myxomatous mitral valve disease

**DOI:** 10.1111/jvim.15854

**Published:** 2020-07-20

**Authors:** Megan H. Poad, Timothy J. Manzi, Mark A. Oyama, Anna R. Gelzer

**Affiliations:** ^1^ University of Pennsylvania Philadelphia Pennsylvania USA

**Keywords:** canine, echocardiography, myxomatous mitral valve disease, radiograph, vertebral heart size, vertebral left atrial size

## Abstract

**Background:**

Evaluation of left heart size helps determine disease severity in dogs with myxomatous mitral valve disease (MMVD).

**Hypothesis/Objectives:**

Determine the ability of radiographic vertebral heart size (VHS) and vertebral left atrial size (VLAS) to predict LHE_ECHO_ in dogs with preclinical MMVD.

**Animals:**

Seventy client‐owned dogs with MMVD and no historical or present clinical or radiographic evidence of congestive heart failure (CHF).

**Methods:**

Retrospective cross‐sectional study of dogs with same‐day echocardiography and thoracic radiography. Receiver‐operating characteristic (ROC) curves were used to assess the ability of VHS, VLAS, and VHS + VLAS to discern dogs with and without LHE_ECHO_, and clinically relevant cutpoints for these radiographic measurements were selected.

**Results:**

The ability of VHS and VHS + VLAS to predict LHE_ECHO_ was moderate (area under the curve [AUC]_VHS_ = 0.851; 95% CI, 0.762‐0.941; AUC_VHS + VLAS_ = 0.865; 0.783‐0.947), and performance of VLAS and VHS + VLAS was not different from that of VHS alone. A VHS cutpoint of >10.8 had sensitivity = 91.1% (76.3%‐98.1%) and specificity = 69.4% (51.9%‐83.7%) for predicting LHE_ECHO_. A cutpoint of >11.7 had sensitivity = 32.4% (17.4%‐50.5%) and specificity = 97.2% (85.5%‐99.9%) for predicting LHE_ECHO_. Thirty (43%) of the 70 dogs had a VHS value of 10.9 to 11.7.

**Conclusions and Clinical Importance:**

Vertebral heart size >11.7 identified dogs with LHE_ECHO_ and VHS ≤ 10.8 excluded dogs with LHE_ECHO_. A large percentage of dogs had VHS values intermediate to these cutpoints.

**Abbreviations**
Ao: aorta
AUC: area under the curve
LA: left atrium
LHE_ECHO_: echocardiographic left heart enlargement
LVIDDN: body weight‐normalized left ventricular internal diameter in diastole
MMVD: myxomatous mitral valve disease
MR: mitral regurgitation
ROC: receiver operating characteristic
VHS: vertebral heart size
VLAS: vertebral left atrial size

## 1 INTRODUCTION

Myxomatous mitral valve disease (MMVD) is the most common cardiovascular disease in dogs,^1^, ^2^ with higher prevalence in small breed dogs (≤20 kg).^3^ Resultant mitral regurgitation (MR) can lead to cardiac remodeling and dilatation of the left ventricular and left atrial chambers. Evaluation of left heart size in asymptomatic dogs is performed to determine severity of disease and identify dogs that could benefit from medical treatment. Administration of pimobendan to dogs with preclinical MMVD and specific radiographic and echocardiographic evidence of left sided cardiomegaly, including normalized LV internal diameter in diastole (LVIDDN) ≥ 1.7, as measured on M‐mode echocardiogram obtained from the right parasternal short‐axis view, and left atrial to aortic (LA:Ao) ratio ≥ 1.6), as measured from the right parasternal short‐axis 2D view, and 1 radiographic measurement, vertebral heart size (VHS) >10.5, resulted in prolongation of the preclinical period.^4^

Echocardiography is considered the gold standard for evaluating left atrial and left ventricular chamber size, however the skill and expertise required to perform and interpret echocardiographic examinations is often not available. In contrast, thoracic radiography is widely available and vertebral heart size (VHS) is a technique used to quantify cardiac size in dogs, including dogs with MMVD.^5^ There are parallel increases in VHS and echocardiographic left atrial and left ventricular dimensions before onset of congestive heart failure dogs with MMVD.^6^, ^7^, ^8^ Vertebral left atrial size (VLAS), a radiographic measurement specifically targeted to reflect left atrial size, is a useful and repeatable predictor of echocardiographically confirmed left atrial enlargement in dogs with MMVD.^9^

The primary objective of this study was to evaluate the ability of VHS and VLAS to predict echocardiographic criteria for left heart enlargement (LHE_ECHO_) in dogs with preclinical MMVD. We hypothesized that radiographic measurements would predict LHE_ECHO_ and would help determine severity of disease in dogs with preclinical MMVD. We aimed to identify clinically relevant values of VHS, VLAS, and sum of VHS + VLAS that predicted presence and absence of LHE_ECHO_.

## 2 MATERIALS AND METHODS

Records of client‐owned dogs with MMVD and no current or previous clinical or radiographic signs of congestive heart failure presenting to Matthew J. Ryan Veterinary Hospital of the University of Pennsylvania from July 2016 to December 2018 were retrospectively reviewed. Dogs were included based on the following criteria: ≥5 years of age, body weight ≤ 15 kg, systolic heart murmur (≥grade 2/6) with maximal intensity over the mitral valve, echocardiographic evidence of MMVD defined as characteristic thickening and prolapse of the mitral valve leaflets and MR on color flow Doppler, and fractional shortening >30%. Dogs with current or previous clinical or radiographic evidence of cardiogenic pulmonary edema, cardiac disease other than MMVD (including congenital abnormalities, severe pulmonary hypertension or right heart disease, pericardial effusion) were excluded. Severe pulmonary hypertension was defined by a Doppler‐derived systolic pressure gradient between the right ventricle and right atrium ≥70 mmHg, and any dog with more than mild right atrial or right ventricular chamber dilatation or concentric hypertrophy based on subjective assessment was excluded. Dogs were ineligible if receiving cardiac medications or parenteral fluid therapy at the time of enrollment. While sedation is not routinely used for echocardiographic and radiographic testing at this institution, if deemed necessary to achieve light sedation, dogs who received butorphanol IV or IM were included in the study.

Each dog underwent standard 2‐D, M‐mode, and Doppler echocardiographic examination (iE33, Philips Healthcare, Andover, Massachusetts) and 3‐view thoracic radiographic study on the day of their examination. Radiographs were evaluated for malpositioning, vertebral abnormalities, and other thoracic abnormalities, which would result in exclusion of that dog from the study. In addition, English bulldogs and French bulldogs were excluded from the study because of the higher likelihood of vertebral abnormalities in these breeds. Echocardiographic examinations and measurements were performed by a board‐certified cardiologist (AR Gelzer or MA Oyama) or a resident under the supervision of a board‐certified cardiologist. The individuals performing the echocardiographic measurements were blinded to the VHS measurement performed by the 1 observer who was blinded to the echocardiographic data. An average of 3 cardiac cycles was used for each measurement. Left ventricular internal diameter at end‐diastole (LVIDD) and left ventricular internal diameter at end‐systole (LVIDS) were measured on 2‐dimensional or 2‐dimensional‐guided M‐mode right parasternal short axis images. End‐diastole of the LV chamber was defined as the internal dimension at the onset of the QRS complex on the echo‐timing ECG. The end‐systolic LV chamber internal dimension was defined as the minimum chamber dimension. The measurements were made from inner edge (blood‐tissue interface) to inner edge. LVIDD was normalized to body size (LVIDDN) as previously described.^10^ Left atrial and aortic root dimensions were measured by the 2‐D right parasternal short axis view as previously described^11^ and normalized to body weight by the following formula: normalized LAD (LADN) = LAD (cm)/BW (kg)^.355.^12^ Normalized AoD (AoDN) = AoD (cm)/BW (kg)^.341.^10^ LA:Ao ratio (LA:Ao) was calculated from left atrial and aortic root diameters. Left atrial and aortic root dimensions were measured from inner edge to inner edge, timed after the end of the T wave, in the earliest frame in which the aortic valve cusps were closed.

Radiographic VHS was measured with digital calipers from the right lateral radiographic projection.^5^, ^13^ Specifically, the long‐axis dimension was measured from the ventral border of the largest of the main stem bronchi seen in cross section to the most ventral point of the cardiac apex. The short‐axis dimension was drawn perpendicular to the long‐axis dimension from the caudal border of the cardiac silhouette at the ventral aspect of the caudal vena cava to the cranial border of the cardiac silhouette. All VHS measurements were performed by a single experienced observer (TJ Manzi), who was blinded to signalment and echocardiographic findings, because validation of VHS (assessing interobserver variability of VHS by multiple observers) was not the intent of the study. Vertebral left atrial size (VLAS) was performed by the right lateral radiographic projection for each dog.^9^ Specifically, the length between the center of the most ventral aspect of the carina to the caudal aspect of the left atrium at point of intersection with the dorsal border of the caudal vena cava was measured. A line equal in length to this measurement was drawn from the cranial border of the fourth thoracic vertebrae and extended caudally parallel to the vertebral canal. The VLAS reported was the length of this line in vertebral body units. All radiographic measurements were recorded to the nearest 0.1 vertebrae. Measurements of VLAS were performed by 2 observers. The VLAS measurements from 1 of the 2 observers (TJ Manzi), who was blinded to the echocardiographic findings, were used for the comparison against LHE_ECHO_. VLAS measurements performed by the other observer (MH Poad) were used only to calculate interobserver agreement for the VLAS measurement.

Dogs were divided into 2 groups based on whether or not echocardiographic criteria for left heart enlargement (LHE_ECHO_), namely LVIDDN ≥1.7 and LA:Ao ≥1.6, were met. Dogs in group 1 failed to meet either or both LHE_ECHO_ criteria. Dogs in Group 2 met both LHE_ECHO_ criteria.

### 2.1 STATISTICAL ANALYSIS

Signalment, radiographic, and echocardiographic data was tested for normality by D'Agostino & Pearson tests. Data is shown as mean (SD) or median (interquartile range). Comparisons between groups were performed by unpaired *t*‐tests or Mann‐Whitney *U* tests based on results of normality testing. The diagnostic utility of radiographic measurements to discern between dogs in Group 1 or 2 was assessed by receiver‐operating characteristic (ROC) curves. The area under the curve (AUC), and determination of the sensitivity, specificity, and likelihood ratios (LR) of various values of the variables of interest was determined. We defined AUC values >.85 as indicative of a clinically useful test,^14^ whereas an AUC value of .5 indicates the test is no better than chance. The LR is the probability of a test result in a subject with disease over the probability of the same test result in a subject without disease. Values of LR > 1 indicate an increase in the posttest probability whereas LR values <1 indicate a decrease in the posttest probability. The change in probability is approximated as ln(LR) x .19^15^. Based on this formula, one can approximate likelihood ratios of 2, 4, and 6 as increasing the pretest probability of disease by an absolute value of ∼15%, 25%, and 35%, respectively, whereas likelihood ratios of .5, .3, and .1 decrease pretest probability of disease by an absolute value of ∼15%, 25%, and 45%, respectively.^15^ For instance, if the pretest probability of disease is 50%, a likelihood ratio of 6 increases the posttest probability of disease to ∼85%, whereas a likelihood ratio of .1 decreases the posttest probability of disease to ∼5%. Univariate and multivariate logistic regression was used to calculate the odds ratios associated with radiographic measurements and presence or absence of LHE_ECHO_ or LA:Ao ≥1.6. The level of agreement between 2 observers measuring VLAS was determined by calculation of the interclass correlation coefficient (ICC) by a model of absolute agreement. The level of agreement was described as none, slight, fair, moderate, and substantial for ICC values of 0 to .10, .11 to .40, .41 to .60, .61 to .81, and .81 to 1.0, respectively.^16^ The strength of correlation between LA:Ao and VLAS was determined by Spearman correlation coefficient (*r*_*S*_) and described as very high, high, moderate, low, and negligible, for values of .9 to 1.0, .7 to .9, .5 to .7, .3 to .5, and 0 to .3, respectively.^17^ Significance was defined as *P* < .05. Statistical analysis was performed by commercial software (STATA 14.0, Stata Corp, College Station, Texas; Medcalc 15.6.1, Ostend, Belgium).

## 3 RESULTS

Seventy dogs, including 36 dogs in Group 1 and 34 dogs in Group 2, were enrolled between July 2016 and December 2018. The signalment, heart murmur grade, and radiographic and echocardiographic characteristics of the groups are displayed in Table 1. Mean age, body weight, and AoDN did not differ significantly between groups. Values of VHS, VLAS, LVIDDN, LADN, and LA:Ao ratio in Group 2 were significantly greater than the corresponding values in Group 1. Group 1 consisted of 13 mixed breed dogs, 5 Cavalier King Charles spaniels, 3 Chihuahuas, 3 Dachshunds, 3 Shih Tzus, 2 Havanese, and 1 dog from each of 7 other breeds. Group 2 consisted of 7 Cavalier King Charles spaniels, 6 mixed breed dogs, 4 Chihuahuas, 3 Pomeranians, 2 Cocker spaniels, 2 Shih Tzus, and 1 dog from each of 9 other breeds. Group 1 consisted of 22 males and 14 females. Group 2 consisted of 22 males and 12 females. Of the 36 dogs in Group 1, 7 had a grade II/VI murmur, 10 had a grade III/VI murmur, 16 had a grade IV/VI murmur, and 3 had a grade V/VI murmur. Of the 34 dogs in Group 2, 22 had a grade IV/VI murmur, 11 had a grade V/VI murmur, and 1 had a VI/VI murmur.

**TABLE 1:** Age, body weight, radiographic vertebral heart size (VHS), vertebral left atrial size (VLAS) echocardiographic normalized left ventricular end‐diastolic dimension (LVIDDN), normalized left atrial diameter (LADN), normalized aortic root diameter (AoDN) and left atrial to aortic root ratio (LA:Ao) of 70 dogs with asymptomatic degenerative mitral valve disease

	Group 1	Group 2	*P*‐value
N	36	34	
Age (y)	9.8 (2.5)	9.8 (2.4)	.98
Body weight (kg)	7.3 (3.0)	7.5 (2.9)	.76
VHS	10.5 (0.74)	11.6 (0.77)	<.0001
VLAS	2.2 (0.32)	2.6 (0.38)	<.0001
LVIDDN	1.5 (1.4‐1.6)	1.9 (1.8‐2.0)	<.0001
LADN	0.99 (0.95‐1.1)	1.4 (1.2‐1.6)	<.0001
AoDN	0.75 (0.08)	0.72 (0.08)	.14
LA:Ao	1.4 (1.3‐1.5)	2.0 (1.8‐2.1)	<.0001
*Heart murmur grade*			
II/VI	7	0	
III/VI	10	0	
IV/VI	16	22	
V/VI	3	11	
VI/VI	0	1	

**FIGURE 1:**
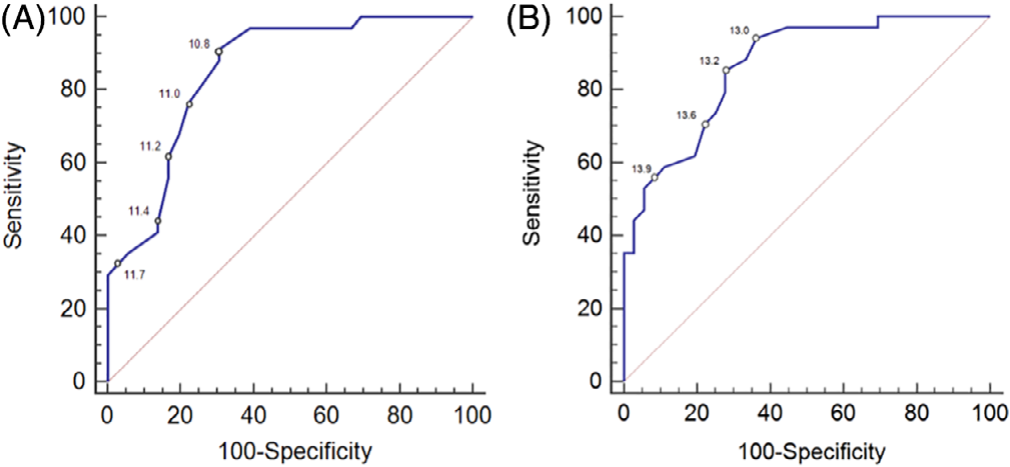
Receiver‐operating characteristic curve of A, radiographic vertebral heart size (VHS) and B, the sum of VHS and radiographic vertebral left atrial size (VLAS) to differentiate dogs with normalized echocardiographic left ventricular internal dimension ≥1.7 and left atrial to aortic root ratio ≥ 1.6. The values of various VHS and VHS + VLAS cutpoints along their respective curves are indicated

**FIGURE 2:**
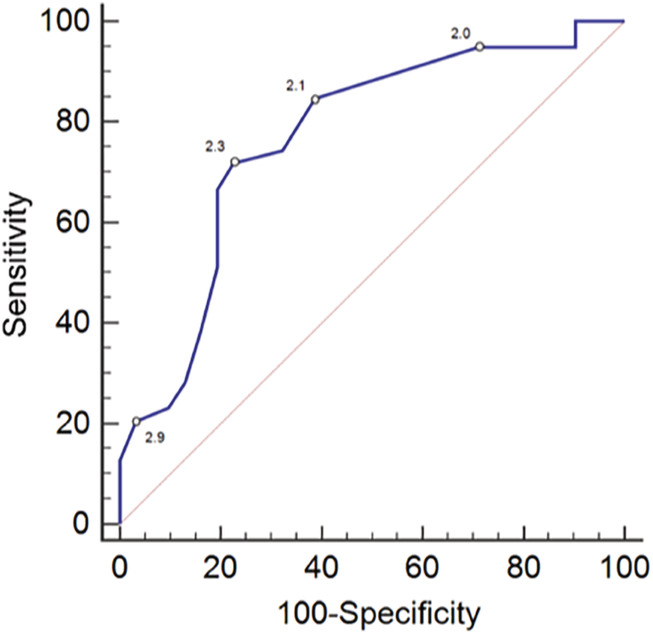
Receiver‐operating characteristic curve of radiographic vertebral left atrial size (VLAS) to differentiate dogs with left atrial to aortic root ratio ≥ 1.6. The values of various VLAS cutpoints along their respective curves are indicated

The discriminatory abilities of VHS and VHS + VLAS to detect LHE_ECHO_ were clinically useful, with AUC of the ROC curve of .851 (95% CI, .762‐.941, Figure 1A) and .865 (95% CI, .783‐.947, Figure 1B), respectively (Figure 1). There was no significant difference in AUC values between the 2 radiographic measurements (*P* = .59). The discriminatory ability of VLAS to detect LA:Ao ≥1.6 was not clinically useful with AUC of the ROC curve of .767 (95% CI, .652‐.883) (Figure 2). The sensitivity, specificity, and positive and negative likelihood ratios with 95% confidence intervals of select values of VHS, VHS + VLAS, and VLAS are displayed in Table 2. Two different VHS cutpoints appeared clinically useful. Dogs with VHS ≤10.8 were associated with a LR of .13, which indicated an absolute decrease in the posttest probability of LHE_ECHO_ of −38.8%. Thus, in a dog whose pretest probability of LHE_ECHO_ was 50%, a VHS of ≤10.8, reduced the probability to 11.2%. Dogs with VHS >11.7 were associated with a LR of 11.7, which indicated an absolute increase in the posttest probability of LHE_ECHO_ of +46.7%. Thus, in a dog whose pretest probability of LHE_ECHO_ was 50%, a VHS of >11.7, increased the probability to 96.7%. Univariate logistic regression revealed that for every .25 increase in VHS, the odds ratio associated with presence of LHE_ECHO_ increased by 1.79 (95% CI, 1.33‐2.40; *P* < .0001). There were 30/70 (42.9%) dogs, including 10/36 dogs (27.8%) in Group 1 and 20/34 dogs (58.8%) in Group 2 possessing VHS between 10.9 and 11.7, which made detection of LHE_ECHO_ in this cohort by VHS difficult.

**TABLE 2:** Sensitivity, specificity, and positive (+LR) and negative likelihood ratios (−LR) of various radiographic vertebral size (VHS) and sum of VHS and vertebral left atrial size (VLAS) cutpoints for determination of normalized echocardiographic left ventricular internal dimension ≥1.7 and left atrial to aortic root ratio ≥ 1.6. The 95% confidence intervals are displayed below the main values

Parameter	Sens (%)	Spec (%)	+LR	−LR
*VHS*				
*To discriminate LA*:*Ao ≥ 1.6 and LVIDDN ≥ 1.7*				
>10.8	91.1	69.4	2.98	.13
	76.3‐98.1	51.9‐83.7	1.8‐4.9	.04‐.4
>11.2	61.8	83.3	3.71	.46
	43.6‐77.8	67.2‐93.6	1.7‐8.1	.3‐.7
>11.7	32.4	97.2	11.7	.70
	17.4–50.5	85.5‐99.9	1.6‐85.4	.5‐.9
*VHS + VLAS*				
*To discriminate LA*:*Ao ≥ 1.6 and LVIDDN ≥ 1.7*				
>13.0	94.2	63.9	2.61	.092
	80.3‐99.3	46.2‐79.2	1.7‐4.1	.02‐.4
>13.6	70.6	77.8	3.18	.38
	52.5‐84.9	60.8‐89.9	1.7‐6.1	.2‐.7
>13.9	55.9	91.7	6.71	.48
	37.9‐72.8	77.5‐98.2	2.2‐20.6	.3‐.7
*VLAS*				
*To discriminate LA*:*Ao ≥ 1.6*				
>2.0	94.9	29.0	1.34	.18
	82.7‐99.4	14.2‐48.0	1.1‐1.7	.04‐.8
>2.3	71.8	77.4	3.18	.36
	55.1‐85.0	58.9‐90.4	1.6‐6.3	.2‐.6
>2.9	20.5	96.8	6.36	.82
	9.3‐36.5	83.3‐99.9	.8‐48.2	.7‐1

Various VLAS‐related cutpoints appeared clinically useful. Univariable logistic regression revealed that for every .25 increase in VHS + VLAS the odds associated with presence of LHE_ECHO_ increased by 1.59 (95% CI, 1.27‐1.99; *P* < .0001). A total of 23/70 dogs (33%) including 10/36 dogs (28%) in Group 1 and 13/34 dogs (38.2%) in Group 2 had a VHS + VLAS value from 13.1 to 13.9. Univariable logistic regression revealed that for every .1 increase in VLAS the odds associated with presence of LA:Ao ≥1.6 increased by 1.32 (95% CI, 1.12‐1.55; *P* = .001). We utilized a multivariable logistic model containing both VHS and VLAS to further explore prediction of LHE_ECHO_, the results of which indicated that only VHS was independently associated with LHE_ECHO_. After adjusting for VLAS, for every .25 increase in VHS, the odds ratio associated with LHE_ECHO_ increased by 1.64 (95% CI, 1.20‐2.25; *P* = .002). A multivariable model that included VHS and VHS + VLAS was not stable because of multicollinearity between the independent variables. For the cohort of 30 dogs with indiscriminate VHS values between 10.9 and 11.7, the utility of subsequent evaluation by VHS + VLAS was not clinically useful (AUC = .533; 95% CI, .312‐.753).

**FIGURE 3:**
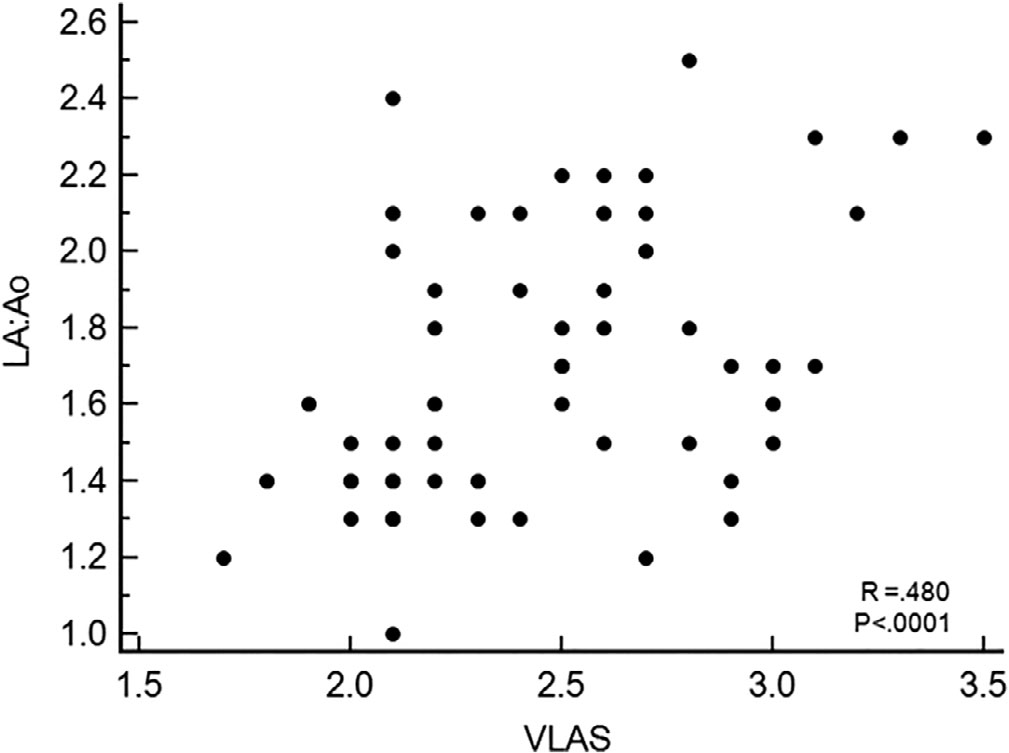
Correlation between radiographic vertebral left atrial size (VLAS) and echocardiographic left atrial to aortic root dimension ratio (LA:Ao)

Level of agreement between the 2 observers for measurement of VLAS was substantial with ICC of 0.932 (95% CI, .901‐.961). Correlation between LA:Ao and VLAS was low (*R*_*s*_ = .480; *P* < .0001) (Figure 3).

## 4 DISCUSSION

Our study results indicated that 2 different VHS values were useful for differentiating presence or absence of LHE_ECHO_ in dogs with preclinical MMVD. Dogs with VHS ≤10.8 were unlikely to possess LHE_ECHO_. This cutpoint was associated with a low number of false negative results, with only 3 of the 34 dogs with LHE_ECHO_ having a VHS ≤10.8. The VHS value of 10.8 is similar to the upper reference value of VHS of 10.7 for various breeds,^5^ however, certain breeds, including Bulldogs, Pugs, Boston terriers, Whippets, and Cavalier King Charles spaniels, have an upper reference value that exceeds 10.8,^18^, ^19^, ^20^ which limits the usefulness of this cutpoint presumably because of false positive results. Our study population included a substantial number of Cavalier King Charles spaniels, and further study is required to determine the utility of breed‐specific cutpoints of VHS for identifying LHE_ECHO_ in dogs with MMVD. Ninety‐one percent of dogs with a VHS >11.7 possessed LHE_ECHO_ and the posttest probability of LHE_ECHO_ increased by an absolute value of 47%. Thus, considering the breed variations in normal VHS, the cutpoint value of >11.7 might be most useful for identifying LHE_ECHO_ in certain dog breeds such as in Cavalier King Charles spaniels with preclinical MMVD. In dogs whose pretest probability of presence or absence of LHE_ECHO_ is equal (ie, 50:50) a VHS of >11.7 increases the probability of LHE_ECHO_ to nearly 100%. Our results agree with a previous retrospective study of dogs with preclinical MVVD that reported VHS >11.7 reliably identified LHE_ECHO_ with high specificity (Vitt JP, Gordon S, Fries RC, et al. Utility of VHS to predict echocardiographic EPIC trial inclusion criteria in dogs with myxomatous mitral valve disease: a retrospective multicentre study [abstract]. ECVIM‐CA Congress; 2017; Saint Julian's, Malta). Importantly, the suggested VHS cutpoint of >11.7 is less than the median VHS (12.5) of a large population of dogs of various breeds with congestive heart failure secondary to MMVD.^21^ A significant limitation of the use of VHS for the purpose of predicting LHE_ECHO_ is the substantial proportion of dogs in our population (30/70 dogs [42.9%]) with VHS values between 10.9 and 11.7, for which VHS alone was not sufficient to accurately identify LHE_ECHO._ Subsequent evaluation of VLAS did not significantly improve detection of LHE_ECHO_ in this cohort. This limitation is clinically important insofar as many dogs with preclinical MMVD possess VHS values in an intermediate range. As an example, a previous study reported that 84/247 (34%) dogs with preclinical MMVD had VHS values between 10.6 and 11.7 (Vitt JP, Gordon S, Fries RC, et al. Utility of VHS to predict echocardiographic EPIC trial inclusion criteria in dogs with myxomatous mitral valve disease: a retrospective multicentre study [abstract]. ECVIM‐CA Congress; 2017; Saint Julian's, Malta).

Another important finding of the current study involves VLAS, which is a potential quantitative method to estimate echocardiographic LA size and to predict echocardiographic LA enlargement in dogs with various degrees of MMVD severity.^9^ Our results indicated that VLAS values ≤2.0 and >2.9 were associated with a high sensitivity and specificity, respectively, for identifying dogs with an LA/Ao >1.6. These results are in agreement with a previous study^9^ of 103 dogs with preclinical MMVD that reported similar VLAS values of ≤1.8 and ≥2.8, however, in the current study, results of logistic regression and ROC analysis indicated that compared to by VHS alone, neither VLAS or VHS + VLAS significantly improved detection of LHE_ECHO._ In our cohort, the correlation between VLAS and LA:Ao was relatively low (*r*_*s*_ = .48) and considerably below a previously reported value of .70.^9^ The reason for this discrepancy is not clear and could be because of differences in population of dogs. For instance, the previous study^9^ included not only dogs with preclinical MMVD but also healthy dogs and dogs with congestive heart failure, and prediction of LA:Ao by VLAS in these latter populations with either normal or markedly enlarged atrial dimensions might be more accurate.

Our study has several limitations. Although VHS has been previously shown to correlate well with echocardiographic measures, obtaining an accurate value and being able to utilize a VHS cutpoint as a diagnostic tool depends on many factors, including positioning of the dogs body, presence of concurrent thoracic abnormalities, radiographic technique, and the observer's level of experience and confidence in the measurement obtained. We aimed to control for some of these variables by performing measurements by only a right lateral projection of radiographs without gross malpositioning of the of the dogs body or clinically important concurrent thoracic abnormalities and by measurements performed by an experienced observer familiar with the anatomic landmarks of VHS measurements. However, all of the aforementioned factors must be considered when utilizing this type of radiographic measurement for diagnostic purposes. Although the use of 1 individual performing VHS measurements was intentional for the goals of the study, it is possible that the selected VHS cutpoints were influenced by this 1 observer's level of experience, expertise, or systematic method of measurement. Additionally, increased VHS for reasons other than left heart enlargement because of MMVD, including right heart enlargement, cardiac tumors, and pericardial effusion will also confound results, however, these conditions were excluded in the current study. As previously mentioned, normal reference ranges for VHS can be different between breeds, including some highly predisposed to MMVD, such as the Cavalier King Charles spaniel, and breed‐specific VHS criteria to identify LHE_ECHO_ warrants further study.

It should be emphasized that the clinical use of the results of this study apply only to dogs with MMVD. The ability of VHS and VLAS to predict LHE_ECHO_ in dogs with other forms of cardiac disease, such as dilated cardiomyopathy and congenital cardiac disease, was not evaluated in the present study and necessitates further research.

In summary, the radiographic VHS is clinically useful in identifying LHE_ECHO_ in dogs with preclinical MMVD, but is limited by a substantial proportion of dogs with indeterminate VHS values. Radiographic measures of LA size such as VLAS possessed low correlation to echocardiographic LA size and did not significantly contribute to detection of LHE_ECHO_ in comparison to use of VHS alone in dogs with preclinical MMVD.

## CONFLICT OF INTEREST DECLARATION

Authors declare no conflict of interest.

## OFF‐LABEL ANTIMICROBIAL DECLARATION

Authors declare no off‐label use of antimicrobials.

## INSTITUTIONAL ANIMAL CARE AND USE COMMITTEE (IACUC) OR OTHER APPROVAL DECLARATION

Authors declare no IACUC or other approval was needed.

## HUMAN ETHICS APPROVAL DECLARATION

Authors declare human ethics approval was not needed for this study.
